# Prevalence, Awareness, Treatment and Influence of Socioeconomic Variables on Control of High Blood Pressure: Results of the ELSA-Brasil Study

**DOI:** 10.1371/journal.pone.0127382

**Published:** 2015-06-23

**Authors:** Dóra Chor, Antonio Luiz Pinho Ribeiro, Marilia Sá Carvalho, Bruce Bartholow Duncan, Paulo Andrade Lotufo, Aline Araújo Nobre, Estela Mota Lima Leão de Aquino, Maria Inês Schmidt, Rosane Härter Griep, Maria Del Carmen Bisi Molina, Sandhi Maria Barreto, Valéria Maria de Azeredo Passos, Isabela Judith Martins Benseñor, Sheila Maria Alvim Matos, José Geraldo Mill

**Affiliations:** 1 Department of Epidemiology, National School of Public Health, Oswaldo Cruz Foundation, Rio de Janeiro, RJ, Brazil; 2 Department of Internal Medicine, Federal University of Minas Gerais, Belo Horizonte, MG, Brazil; 3 Scientific Computing Program, Oswaldo Cruz Foundation, Rio de Janeiro, RJ, Brazil; 4 Postgraduate Studies Program in Epidemiology, School of Medicine, Federal University of Rio Grande do Sul, Porto Alegre, RS, Brazil; 5 School of Medicine, University of Sao Paulo, São Paulo, SP, Brazil; 6 Institute of Collective Health, Federal University of Bahia, Salvador, BA, Brazil; 7 Laboratory of Health and Environment Education, Oswaldo Cruz Foundation, Rio de Janeiro, RJ, Brazil; 8 Post Graduate Program of Collective Health, Federal University of Espírito Santo, Vitória, ES, Brazil; 9 Faculty of Medicine, Federal University of Minas Gerais, Belo Horizonte, MG, Brazil; 10 Department of Physiological Sciences, Federal University of Espirito Santo, Vitória, ES, Brazil; University of South Carolina, UNITED STATES

## Abstract

High blood pressure (HBP) is the leading risk factor for years of life lost in Brazil. Factors associated with HBP awareness, treatment and control need to be understood better. Our aim is to estimate prevalence, awareness, and types of anti-hypertensive treatment and to investigate the association of HBP control with social position. Data of 15,103 (54% female) civil servants in six Brazilian state capitals collected at the Brazilian Longitudinal Study of Adult Health (ELSA-Brasil) baseline (2008-2010) were used to estimate prevalence and cross-sectional association of HBP control with education, per capita family income and self-reported race, using multiple logistic regression. Blood pressure was measured by the oscillometric method. 35.8% were classified as presenting HBP; 76.8% of these used anti-hypertensive medication. Women were more aware than men (84.8% v. 75.8%) and more often using medication (83.1% v. 70.7%). Adjusted HBP prevalence was, in ascending order, Whites (30.3%), Browns (38.2%) and Blacks (49.3%). The therapeutic schemes most used were angiotensin-converting enzyme inhibitors, in isolation (12.4%) or combined with diuretics (13.3%). Among those in drug treatment, controlled blood pressure was more likely in the (postgraduate) higher education group than among participants with less than secondary school education (PR = 1.21; 95% CI: 1.14–1.28), and among Asian (PR = 1.21; 95% CI: 1.12–1.32) and ‘Whites (PR = 1.19; 95% CI: 1.12–1.26) compared to Blacks. Socioeconomic and racial inequality—as measured by different indicators—are strongly associated with HBP control, beyond the expected influence of health services access.

## Introduction

High blood pressure (HBP) accounted for 9.4 million deaths and 7% of global disability-adjusted life years in 2010, making it the leading single risk factor in the global burden of disease[1]. According to recent World Health Organization (WHO) estimates, the prevalence of HBP in adults (≥25 years) is 29.2% of males and 24.8% of females[2], leading to worldwide prevalence of hypertension estimated at more than 1 billion individuals. About 54% of strokes and 47% of coronary heart disease worldwide are attributable to HBP[3], which is also a risk factor for heart failure, diabetes, chronic kidney disease, cognitive decline and other diseases. Overall, about 80% of the HBP-related burden of disease occurs in low-income and middle-income countries[3], where the prevalence of hypertension has been rising and rates of awareness, treatment and control are lower than in developed countries[4].

Brazil is a South American country of continental dimensions with the fifth-largest population in the world, where rates of morbidity and mortality are strongly affected by geographical, racial and social inequalities[5]. Cardiovascular diseases, particularly stroke and coronary heart disease, have been–and, despite their decline, continue to be–the main cause of death in Brazil[6]: in 2010, about 29% of all deaths were attributable to cardiovascular diseases[7]. HBP is the leading risk factor for death and years of life lost in Brazil[1]. Nonetheless, information about prevalence, awareness, treatment and control of HBP in Brazil is limited[8–10]. National prevalence estimates are derived from interviews and telephone surveys[11,12] and data about HBP awareness, treatment and control are available only from local surveys conducted in specific Brazilian cities or states[13–16]. Moreover, no studies of antihypertensive drug prescribing patterns in Brazil were found. Accurate data on prevalence, awareness, management and control from a larger, more diverse Brazilian sample can guide future evidence-driven health policies and may allow comparisons with other countries[17].

In Brazil, it is largely unknown how socioeconomic, racial and demographic factors influence control of HBP. Higher prevalence of hypertension and poorer therapeutic control have been noted among those of lower socioeconomic position[18,19]. Social class, race and gender are the basic axes of the social hierarchy which shape "the causes of causes"[20] of diseases, since they cause an uneven distribution of risk factors, including HBP[21]. Examining differences in the prevalence and control of HBP by education, income, race and gender helps avoiding the oversimplification of the multifaceted nature of social disadvantage and its effects on health[22].

ELSA-Brasil (*Estudo Longitudinal de Saúde do Adulto*—Longitudinal Study of Adult Health) is a large (15,105-subject), multiracial, multicentre cohort study focused on the risk of cardiovascular diseases, diabetes, and obesity in Brazilian adults aged 35–74 years[23]. From the ELSA cohort’s baseline data, this present study examined prevalence, awareness, management and control of HBP, as well as patterns of antihypertensive drug use. It further evaluated, in participants using antihypertensive drugs, how controlled high blood pressure associated with age, gender, education levels, per capita family income and race.

## Methods

### Study population

Detailed information on ELSA-Brasil can be found in another publication[23]. Briefly, it is a multicentre cohort study involving public servants at six teaching and research institutions in six towns in Brazil. Its main aims were to examine the incidence of cardiovascular disease and diabetes, as well as their main social, contextual, occupational and biological determinants. Baseline assessment consisted of an approximately 7-hour evaluation, which included a comprehensive set of measurements, clinical, laboratory and imaging exams and a detailed in-person interview by trained personnel.

For the baseline study (2008–2010), 15,105 retired and active civil servants from 35 to 74 years old were recruited, out of which 2 individuals were excluded because of there was no information on anti-hypertensive drug use.

### Study variables

#### Measurement of blood pressure and anti-hypertensive drugs

Arterial pressure was measured after five minutes’ rest[24], with the participant sitting in a quiet room at controlled temperature (20–24°C), using a validated oscillometric device (Omron HEM 705CPINT)[24,25], following recommendations of the Seventh Report of the Joint National Committee on Prevention, Detection, Evaluation, and Treatment of High Blood Pressure (2004)[26]. Three measurements were taken at one-minute intervals[27].

Quality assurance and control procedures were implemented across centres to minimise error. Test-retest measurements were performed under similar conditions shortly after the original set of measurements. The intra-class correlation coefficients were 0.88 (95% CI 0.82;0.91) for systolic blood pressure and 0.89 (95% CI 0.83;0.82) for diastolic blood pressure[28].

All participants were asked about use of continuous medication in the prior two weeks[29] and were instructed to bring prescriptions and/or drugs used to the study clinic. This information was recorded during the interview on a specifically-prepared form. The antihypertensive drugs reported by participants were classified into seven categories by main pharmacological action: diuretics (thiazides, loop diuretics, aldosterone antagonists and potassium-sparing drugs); beta-blockers; calcium-channel blockers; angiotensin-converting enzyme (ACE) inhibitors; angiotensin-II antagonists; vasodilators (direct action); and central and peripheral sympatholytics.

#### Definitions

Casual blood pressure (BP) was considered to be the mean of the last two of the three BP measurements taken. Hypertension was defined in terms of three criteria: systolic BP ≥ 140 mmHg or diastolic BP ≥ 90 mmHg or use of medication to treat HBP.

To be considered an anti-hypertensive drug user a participant had to report a specific medication in at least one of the categories listed above and to respond ‘Yes’ to the question "Are any of the drugs you have taken in the past two weeks for hypertension (high blood pressure)?".Controlled blood pressure was defined as SBP <140 mm Hg and DBP <90 mm Hg.

Those who responded ‘Yes’ to the question "Has a doctor ever told you that you have hypertension (high blood pressure)?" were considered to be aware of the condition of HBP, except women who reported the diagnosis only during pregnancy. Controlled HBP was defined as systolic BP < 140 mmHg and diastolic BP < 90mmHg. This study did not evaluate non-drug treatment of HBPhypertension.

#### Covariates

The following covariates were considered: age (35–44,45–54,55–64,65–74), gender, education (<secondary, secondary, undergraduate, postgraduate), self-declared race (Black, Brown, White, Asian and Indigenous) and per capita family income in US dollars (USD) (<500.00, 501.00 to 1000.00, >1000.00)—local currency, Brazilian reais (BRL) was converted to USD at a rate of BRL 2.00 = US$1.00.

### Statistical analysis

Cohort characteristics were summarized using prevalence of high blood pressure by age, gender, education, per capita family income and race. Prevalence adjusted for age and gender were estimated using a logistic regression model[30]. It consists in direct adjustment to estimate prevalence ratio from logistic regression using the conditional and marginal methods. Asymptotic confidence intervals for the conditional and marginal prevalence ratios were proposed by Flanders and Rhodes[31]. Details can be found in Bastos et. al 2015[32]. In this paper we estimated the prevalence ratio using marginal methods. The adjusted prevalences were estimated using the same approach. Prevalence of HBP, awareness, anti-hypertensive drug use and controlled HBP were also calculated by age and gender.

The association between covariates and controlled HBP was investigated through multiple logistic regression. We fitted four models including each socio-demographic variable step by step, in order to be able to discuss the contribution and changes of estimates in each one.The Marginal prevalence ratios were estimated as proposed by Wilcosky&Chambless[30]. All analyses were performed using R Core[33].

### Ethical considerations

Approvals were granted by all institutional review boards: Sao Paulo University, Oswaldo Cruz Foundation, Federal University of Bahia, Federal University of Minas Gerais, Federal University of Espírito Santo, and Federal University of Rio Grande do Sul. All participants signed declarations of informed consent.

## Results

Prevalence of HBP was greater among the men than among the women (40.1% vs. 32.2%) and increased with age (Table 1). In addition, it varied strongly and inversely with level of education (prevalences adjusted for sex and age), from 44% among participants who had not completed secondary school to 28.4% among those with postgraduate studies; and also with per capita family income. Participants who classified themselves as Black showed greater adjusted prevalence (49.3%) than those who classified themselves in the other colour/race categories.

**Table 1:** Study population (n and %) and prevalence of high blood pressure by gender, age, socioeconomic position and race. Longitudinal Study of Adult Health (ELSA-Brasil), 2008–2010.

		High blood pressure		
		Crude	Adjusted by age and gender	
Variables	N (%)	N	%	%
**Gender**				
Male	6888 (45.6)	2760	40.1	40.1
Female	8215 (54.4)	2642	32.2	32.2
**Age**				
35 to 44	3341 (22.1)	531	15.9	15.8
45 to 54	5937 (39.3)	1859	31.3	31.3
55 to 64	4235 (28.1)	1994	47.1	47.3
65 to 74	1590 (10.5)	1018	64.0	63.7
**Education**				
<Secondary complete	1922 (12.7)	1012	52.7	44.0
Secondary complete	5233 (34.7)	1998	38.2	40.8
Undergraduate complete	2415 (16.0)	768	31.8	34.9
Postgraduate	5533 (36.6)	1624	29.4	28.4
**Per capita family income (USD)**				
<500.00	5564 (37.0)	2172	39.0	40.9
501.00 to 1000.00	5142 (34.2)	1721	33.5	34.6
>1000.00	4327 (28.8)	1484	34.3	30.7
**Colour/Race**				
Black	2397 (16.1)	1158	48.3	49.3
Brown	4202 (28.2)	1558	37.1	38.2
White	7789 (52.2)	2421	31.1	30.3
Asian	374 (2.5)	126	33.7	32.1
Indigenous	157 (1.0)	60	38.2	34.4

No information: Income (n = 70); Race (n = 184) (excluded from the total).

Overall, 35.8% were classified as having HBP (Table 2 and S1 File). Of these, 80.2% were aware they had HBP, that awareness being more frequent among women than among men (84.8% vs. 75.8%) in all age groups, although especially salient in the youngest age group (35–44 years old).

**Table 2:** Prevalence of high blood pressure, awareness, use of anti-hypertensive drugs and blood pressure control, by gender and age, 2008–2010. Longitudinal Study of Adult Health (ELSA-Brasil), 2008–2010.

Variables	Prevalence of high blood pressure				Aware		Use drugs		Blood Pressure Controlled		
Age (years)	Gender	N	%	Total	Yes	%	N	%	N	% with high blood pressure	% of those using drug(s)
**35 to 44**	M	308	19.7	1563	192	62.3	160	52.0	109	35.4	68.1
	F	223	12.5	1778	178	79.8	162	72.7	123	55.2	75.9
**45 to 54**	M	970	36.2	2682	694	71.6	619	63.8	418	43.1	67.5
	F	889	27.3	3255	743	83.6	718	80.8	568	63.9	79.1
**55 to 64**	M	963	51.9	1856	776	80.6	737	76.5	471	48.9	63.9
	F	1031	43.3	2379	900	87.3	881	85.5	637	61.8	72.3
**65 to 74**	M	519	66.0	787	430	82.9	435	83.8	280	54.0	64.4
	F	499	62.1	803	420	84.2	435	87.2	273	54.7	62.8
**Total**	M	2760	40.1	6888	2092	75.8	1951	70.7	1278	46.3	65.5
	F	2642	32.2	8215	2241	84.8	2196	83.1	1601	60.6	72.9
**Total**		5402	35.8	15103	4333	80.2	4147	76.8	2879	53.3	69.4

Table 2 presents the prevalence of high blood pressure, its awareness and the use of anti-hypertensive drugs among all participants. In addition the proportion of blood pressure control is presented both among all participants classified with high blood pressure and among those using anti-hypertensive drugs.Use of at least one anti-hypertensive drug was reported by 76.8% (n = 4147) of participants classified as having HBP and was also more frequent among women in all age groups (Table 2). Among users of anti-hypertensives, 69.4% showed controlled blood pressure levels (65.5% of the men and 72.9% of the women). Considering all the participants classified as having HBP, about 53% showed appropriate blood pressure levels.

Among those using anti-hypertensives, the prevalence of controlled blood pressure, adjusted for age, level of education, per capita family income and colour/race (Table 3, Model 4), was 15% greater among women than among men (PR = 1.14; 95% CI: 1.10–1.19). Besides, thisprevalence varied inversely with education: among participants with postgraduate studies, it was 21% greater (PR = 1.21; 95% CI: 1.14–1.28) than among those who had not completed secondary school. Interestingly, as compared with those who had not completed secondary school, there was a difference between participants who had postgraduate studies and those with undergraduate studies only: among the latter, the prevalence of controlled blood pressure was smaller (PR = 1.13; 95% CI: 1.06–1.20. Per capita family income showed no statistically significant association in the model adjusted for all the other variables. Prevalence of controlled blood pressure levels was 21% greater among self-reported Asian participants (mainly of Japanese descent) than self-reported Blacks (PR = 1.21; 95% CI: 1.12–1.32), and 19% greater among Whites than among Blacks (PR = 1.19; 95% CI: 1.12–1.26). No difference was observed between Browns and Blacks.

**Table 3:** Association between socioeconomic position, race and control of blood pressure among hypertensives who used anti-hypertensive drugs. Longitudinal Study of Adult Health (ELSA-Brasil), 2008–2010.

	Controlled blood pressure	
	PR	CI (95%)
**Model 1: age and gender**		
Age	0.99	0.99–1.00
Male	**1.00**	-
Female	1.11	1.07–1.16
**Model 2: Model 1 + education**		
Age	0.99	0.99–1.00
Female	1.13	1.09–1.18
<Secondary complete	**1.00**	-
Secondary complete	1.06	1.01–1.12
Undergraduate complete	1.17	1.11–1.24
Postgraduate	1.30	1.24–1.36
**Model 3: Model 2 + income**		
Age	0.99	0.99–1.00
Female	1.13	1.09–1.18
Secondary complete	1.05	1.00–1.11
Undergraduate complete	1.15	1.08–1.22
Postgraduate	1.26	1.19–1.33
Income per capita <500.00 (USD)	**1.00**	-
Income per capita 501.00 to 1000.00 (USD)	1.05	1.00–1.10
Income per capita >1000.00 (USD)	1.05	1.00–1.12
**Model 4: Model 3 + race**		
Age	0.99	0.99–1.00
Female	1.14	1.10–1.19
Secondary complete	1.05	1.00–1.11
Undergraduate complete	1.13	1.06–1.20
Postgraduate	1.21	1.14–1.28
Income per capita 501.00 to 1000.00 (USD)	1.02	0.97–1.08
Income per capita >1000.00 (USD)	1.01	0.95–1.08
Black	**1.00**	-
Brown	1.05	0.99–1.10
White	1.19	1.12–1.26
Asian	1.21	1.12–1.32
Indigenous	1.18	1.04–1.35

N = 4082.

Note: The reference category for each covariate was specified only when first included.

Among the 4147 participants in treatment with drugs, the classes of anti-hypertensives most used, in isolation or combination, were: diuretics (53%); angiotensin-converting enzyme inhibitors (ACE inhibitors) (38.7%); beta blockers (31.7%); angiotensin II receptor antagonists (29.3%); calcium-channel blockers (18.9%); central action sympatholytics (1.71%); alfa-1 blockers (0.51%); vasodilators (0.4%). Among the diuretics (n = 2471), most used were thiazides (85.7%), followed by aldosterone antagonists and potassium-sparing drugs (11.6%) and loop diuretics (2.8%) (data not shown).

Most used in treatment with one or more drug classes (Table 4) were ACE inhibitors combined with diuretics (13.3%) or alone (12.4%), angiotensin II antagonists (11%), diuretics (9.2%) and beta blockers (8.9%).

**Table 4:** Use of anti-hypertensive drugs by drug classes and combinations. Longitudinal Study of Adult Health (ELSA-Brasil), 2008–2010.

Drug classes and combinations	N	%
Angiotensin converting enzyme inhibitors**+**Diuretics	553	13.3
Angiotensin converting enzyme inhibitors	516	12.4
Angiotensin-II antagonists	455	11.0
Diuretics	382	9.2
Beta blockers	367	8.9
Beta blockers + Diuretics	311	7.5
Beta blockers + Angiotensin-converting enzyme inhibitors + Diuretics	151	3.6
Calcium-channel blockers	117	2.8
Beta blockers + Angiotensin-converting enzyme inhibitors	103	2.5
Calcium-channel blockers **+** Angiotensin-converting enzyme inhibitors+ Diuretics	103	2.5
Calcium-channel blockers **+** Angiotensin converting enzyme inhibitors	86	2.1
Calcium-channel blockers+ Diuretics	66	1.6
Beta blockers+ Calcium-channel blockers	50	1.2
Beta blockers + Calcium-channel blockers + Diuretics	43	1.0
Beta blockers + Calcium-channel blockers + Angiotensin-converting enzyme inhibitors	21	0.5
All five groups	2	0.1
Others	821	19.8
**Total**	4147	100.0

The drugs most used alone (Table 5) were hydrochlorothiazide (39.6%), followed by enalapryl (23.4%), atenolol (21.9%), losartan (19.2%), amlodipine (14.2%), captopril (10.7%), chlortalidone (8.5%), and amiloride(5.4%) in association with other diuretics, and propranolol (4.9%). Among diuretics, the most used was hydrochlorothiazide (78%), followed by the loop diuretic furosemide +bumetamide (3%), or spironolactone (2.5%). Chlorthalidone was used by 17% of participants.

**Table 5:** Use of anti-hypertensive drugs. Longitudinal Study of Adult Health (ELSA-Brasil), 2008–2010.

Name	No	Yes	%
Hydrochlorothiazide	2504	1643	39.6
Enalapril	3176	971	23.4
Atenolol	3240	907	21.9
Losartan	3349	798	19.2
Amlodipine	3559	588	14.2
Captopril	3703	444	10.7
Chlorthalidone	3795	352	8.5
Amiloride	3922	225	5.4
Propranolol	3943	204	4.9
Indapamide	3980	167	4.0
Valsartan	3988	159	3.8
Metroprolol	4019	128	3.1
Lisinopril	4050	97	2.3
Nifedipine	4054	93	2.2
Olmesartan	4062	85	2.1
Candesartan	4065	82	2.0
Furosemide	4083	64	1.5
Ramipril	4083	64	1.5
Telmisartan	4087	60	1.5
Spironolactone	4093	54	1.3
Carvedilol	4096	51	1.2
Clonidine	4101	46	1.1
Others	4146	284	6.9

No information: n = 6.

Total users of anti-hypertensives: 4147.

Others(<1%):Aliskiren, Bisoprolol, Bumetanide, Clopamide, Benazepril, Delapril, Hydralazine, Nebivolol, Sotalol, Verapamil, Manidipine, Diltiazem, Felodipine, Fosinopril, Ibesartan, Isradipine, Lacidipine, Lercanidipine, Doxazosin, Methyldopa, Moxonidine, Nadolol, Nitrendipine, Perindopril, Pindolol, Piretanide, Reserpine, Rilmenidine, Triamterene.

## Discussion

In this cohort, from 35 to 74 years old, 35.8% of participants were classified as hypertensive, with greater prevalence among the men. The women showed more frequent prior awareness of their condition of HBP, use of anti-hypertensive drugs and control of blood pressure (BP). Control of BP among users of anti-hypertensive drugs varied inversely with socioeconomic position and was lower among self-reported Black participants. The drug classes most used for treatment were, in decreasing order, diuretics, ACE inhibitors, beta blockers and angiotensin II antagonists. The great majority of participants classified as having HBP used more than one anti-hypertensive drug, particularly ACE inhibitors in combination with diuretics.

The prevalence of HBP found among participants in ELSA-Brasil is greater than the 28.7% reported in a meta-analysis based on 10 Brazilian cross-sectional studies[8], because, on average, the populations evaluated in this meta-analysis are younger. Just as in ELSA-Brasil, in the investigations in this meta-analysis, blood pressure was measured rather than using the self-reported diagnosis.

The estimate given here is also comparable with those found in the United States in 2011–12 (29.1%), England in 2006 (29%) and China in 2011–12 (40%)[34–36]. Self-reported diagnosis of high blood pressure estimated for the population of Brazil’s state capitals (22.7%) is smaller than the 35.8% found in ELSA, possibly because it includes individuals 18 or more years old[11].

In this study population, 80.2% of participants classified as having high blood pressure were aware of their condition, which is similar to levels in Canada (82.6%) and the United States (74%) in comparable populations [37,38], in spite of the existing differences in health systems, conceptualized in this study as mediators between distal social determinants and blood pressure awareness and control. Consistent with the available literature, awareness of high blood pressure, use of drugs and control of BP were greater among women than men. That result is found in countries as different, socially and culturally, as India, United States, China, South Africa and Cuba [34,39–43]. More frequent use of medical care and health services by women seems to be one of the reasons for that result, reflecting different ways that men and women express disease[44]. Although high levels of awareness are as desirable among men as among women, this ‘advantage’ of women’s has significant impact on overall cardiovascular health, because high blood pressure, diabetes and their combinations have greater impact on cardiovascular risk among women than among men, especially after menopause[45,46].

Importantly, although awareness of a diagnosis of high blood pressure is a key factor in controlling the condition, it is not enough to assure adherence to treatment nor to change behaviour, especially because high blood pressure is generally asymptomatic. Confidence in the health system[47], routine visits to the same service or doctor[48] and the number of visits[49] have been cited as factors that can influence control of high blood pressure. In Brazil, although many of the drugs used are distributed free of charge by the national health system (e.g., all the diuretics most used by the ELSA population, except amiloride), these drugs are not always available. When they have to be paid for, their prices are a considerable constraint on treatment adherence.

Anti-hypertensive drugs were widely used, at levels similar to those reported for the populations of the United States (75.7%) and Canada (79.0%)[34]. The anti-hypertensive drugs most used were thiazide diuretics, which agrees with the recommendation to prefer this drug class by Brazilian and international guidelines current at the time of the study[1,2]. In Brazil, as in other countries, hydrochlorothiazide forms part of most combination antihypertensive pills containing a diuretic. However, there is evidence to suggest chlorthalidone is a much more effective diuretic[50]. Almost all the drugs in use (98%) belong to the five preferred classes (diuretics, ACI inhibitors, beta blockers, angiotensin II antagonists, calcium-channel blockers)[2]. Also noteworthy is that almost 60% of participants used two or more drugs, corroborating evidence that prescribing of multiple drugs is most common for controlling high blood pressure[2].

Controlled blood pressure in about 50% of hypertensives is in line with the literature[37]. Less frequent, however, is the high proportion (about 70%) of our study participants who were in treatment with drugs and whose blood pressure was controlled[38]. In a meta-analysis based on Brazilian cross-sectional studies (10 studies)[8], the percentage of control estimated for Brazil in the 2000s was 24.1%, which is comparable with other countries[49,51]. The difference between control in the ELSA population and in other population groups in Brazil can be explained by the high level of education in our study population. Travassos et al. found that health service use in Brazil can largely be explained in terms of education, income and occupation[44]. As the ELSA population comprises individuals in employment and with high levels of education, socioeconomic position is an important factor conditioning high levels of awareness, treatment and control. In addition, non-drug measures to reduce body weight, particularly leisure-time physical activities, can mediate relations between socioeconomic position and blood pressure control, which would contribute to explaining these good results. Note, however, that prevalence of obesity is really high in ELSA-Brasil, while prevalence of physical activity regarded as sufficient for disease prevention purposes is low[52], suggesting that proper use of drugs may be the most important factor explaining the blood pressure control rates observed.

Despite the socioeconomic position characteristics of the ELSA population and the narrower range of socioeconomic variation than for the overall population of Brazil, unequal prevalence of controlled blood pressure among users of anti-hypertensives are expressed strongly and clearly. As in other studies[41,53,54], participants with less education and, independently, Blacks and Browns show lower frequencies of HBP control[37].

The lower percentage of control among participants with undergraduate, as compared with postgraduate, university education is particularly striking. Given that these two groups have both studied at university level, it is improbable that differences in information as to the importance of control, nor health service use, contributed to that result. However, it is conceivable that greater autonomy and acquisition of new skills (control over work processes), which are identified as protective against men’s developing high blood pressure[55], may partly explain these results. In addition, it can be speculated that this advantageous situation originating in the work environment, coupled with greater social prestige, may be reflected in the way the individual deals with health-related factors overall.

The association between lower socioeconomic position and greater prevalence of HBP is reported in other countries[19,39,53][53], as is the association with lower frequency of awareness, treatment and control[56,57]. Lack of material resources is the source of a vicious circle comprising disadvantages that start during intrauterine life and continue later through less access to healthy foods, less time to engage in, and less access to settings that encourage, physical activity (a habit that is important to preventing or controlling HBP)[58], and less frequent use of health services[44].

In the United States, the excess of high blood pressure shown by Afro-Americans as compared with Whites has been one of the main characteristics of health inequalities[37,53]. Explanatory mechanisms have been polarised between social and genetic factors and it is only recently that racial inequality in the occurrence of cardiovascular diseases has been addressed in epigenetic terms. On that approach, observed biological variations should not be confused with genetic explanations, which are inappropriate. Kuzawa&Sweet[59] write: "A genetic interpretation of the residual race effect problematically conflates observed biological variation with inferred genetic contributions, and ignores evidence that social factors can have durable life-course and transgenerational effects on health. Whereas group membership and continental race are poor predictors of genetic variation, these same categories are directly related to the social and structural manifestations of inequality that impact the development of responsive biological systems".

In addition to showing higher prevalence of HBP, Afro-Americans displayed lower blood pressure control rates, even comparing only those who use anti-hypertensive drugs[37,54]. Socioeconomic position, drug type and cardiovascular comorbidities have been proposed as possible explanations[54]. Racial discrimination has also been investigated as a contributing factor in chronic stress and greater prevalence of HBP[60], especially among Afro-Americans[61].

In Brazil, empirical studies of racial inequalities in the occurrence and control of HBP are scarce, but suggest greater prevalence among Blacks [62–64] and among Blacks who report having suffered discrimination[65].

In ELSA, Black participants represented 25% of the group with up to secondary education and only 6% of those with postgraduate education. In addition, they reported discrimination more often than participants self-reported as in other racial categories (results not presented). It can be imagined that racism and socioeconomic disadvantage act simultaneously to increase the risk of high blood pressure and decrease the opportunities to control it.

This study presents results based on standardised blood pressure measurements and detailed data on anti-hypertensive drug use among 15103 participants of the ELSA-Brasil cohort. As that study was conducted in six of Brazil’s state capitals, these results offer limited scope for generalisation to Brazil’s overall adult population. Although relating to a specific population, the ELSA-Brasil results may indicate the situation of the portion of Brazil’s population comprising residents of large urban centres who are in employment. For example, lower frequencies of HBP awareness and control among men—a finding encountered universally in the available literature–constitute one important bottleneck for prevention and control of HBP in Brazil and other countries. Improved understanding of the different social roles, opportunities and constraints experienced in different manners by men and women can inform public health policies more effectively[66] and contribute to improving men’s health and life expectancy. Besides gender, other fronts on which to extend prevention and control of HBP in Brazil include reducing social inequalities in access to information, use of quality health services and access to public spaces and conditions that facilitate healthy lifestyle habits. Reverse causality, although always possible in cross-sectional analyses, is improbable in interpreting these associations between indicators of social position and control of HBP, because they involve a population of 35 to 74 years old with stable employment, admission to which was unrelated to blood pressure levels.

To conclude, higher levels of HBP awareness and control is an essential precondition to reducing the impact of diseases, complications and deaths from cardiovascular diseases and diabetes in Brazil, which is going through a period of rather peculiar social and demographic transitions. The results of this study and future analyses of the ELSA population, which–unlike other cohorts comprising exclusively individuals with high blood pressure–is made up of workers[67], can yield important contributions to improving understanding of the factors that act to increase the prevalence of high blood pressure and hinder its control. Explanations that extend beyond the health care system can contribute to the development of strategies better tailored to the population and its various constituent groups.

## Supporting Information

S1 File Mean and standard deviation of systolic blood pressure and diastolic blood pressure: according to age groups and sex. (DOCX)
