# Supplementary material for: Prevalence, Awareness, Treatment and Influence of Socioeconomic Variables on Control of High Blood Pressure: Results of the ELSA-Brasil Study
Source: PLoS One. 2015 Jun 23;10(6):e0127382. doi: 10.1371/journal.pone.0127382 (PMC4478044; doi:10.1371/journal.pone.0127382)
Supplement: S1 File — (DOCX) [file pone.0127382.s001.docx]

Appendice 1

Mean and standard deviation of systolic blood pressure and diastolic blood pressure according to age groups and sex. Longitudinal Study of Adult Health

(ELSA-Brasil), 2008-2010.

| **MEN** | **Systolic Blood Pressure (mmHg)** | | | **Diastolic Blood Pressure (mmHg)** | | |
| --- | --- | --- | --- | --- | --- | --- |
|  | sample size | mean | standard deviation | sample size | mean | standard deviation |
| **All ages (years)** | 6888 | 125.57 | 16.82 | 6888 | 78.90 | 10.84 |
| 35-39 | 537 | 119.70 | 11.60 | 537 | 76.51 | 9.42 |
| 40-44 | 1026 | 120.34 | 12.94 | 1026 | 77.42 | 10.36 |
| 45-49 | 1417 | 123.04 | 14.37 | 1417 | 79.16 | 10.32 |
| 50-54 | 1265 | 126.36 | 17.04 | 1265 | 80.90 | 11.18 |
| 55-59 | 1115 | 127.91 | 18.20 | 1115 | 80.12 | 11.53 |
| 60-64 | 741 | 129.30 | 18.35 | 741 | 79.04 | 10.64 |
| 65-69 | 432 | 131.04 | 19.12 | 432 | 77.46 | 11.46 |
| 70-74 | 355 | 135.15 | 20.64 | 355 | 76.27 | 10.48 |
| **WOMEN** | **Systolic Blood Pressure (mmHg)** | | | **Diastolic Blood Pressure (mmHg)** | | |
|  | sample size | mean | standard deviation | sample size | mean | standard deviation |
| **All ages (years)** | 8215 | 117.67 | 16.82 | 8215 | 74.03 | 10.84 |
| 35-39 | 618 | 108.27 | 11.06 | 618 | 70.82 | 8.84 |
| 40-44 | 1160 | 110.73 | 13.10 | 1160 | 72.18 | 9.83 |
| 45-49 | 1698 | 114.29 | 14.47 | 1698 | 73.89 | 10.28 |
| 50-54 | 1557 | 117.31 | 15.44 | 1557 | 74.93 | 10.12 |
| 55-59 | 1448 | 119.96 | 17.09 | 1448 | 75.05 | 10.46 |
| 60-64 | 931 | 124.91 | 18.78 | 931 | 75.24 | 10.13 |
| 65-69 | 546 | 129.49 | 18.74 | 546 | 74.65 | 10.04 |
| 70-74 | 257 | 131.85 | 19.40 | 257 | 74.09 | 10.48 |
